# Advanced adrenocortical carcinoma successfully treated with gemcitabine plus capecitabine as second‐line chemotherapy

**DOI:** 10.1002/iju5.12214

**Published:** 2020-08-30

**Authors:** Akinaru Yamamoto, Yasutomo Nakai, Toshiki Oka, Tomohiro Kanaki, Yoshiyuki Yamamoto, Akira Nagahara, Masashi Nakayama, Ken‐ichi Kakimoto, Kazuo Nishimura

**Affiliations:** ^1^ Department of Urology Osaka International Cancer Institute Osaka City Osaka Japan

**Keywords:** adrenocortical carcinoma, capecitabine, chemotherapy, gemcitabine, second‐line chemotherapy

## Abstract

**Introduction:**

Adrenocortical carcinoma is a rare malignant tumor with an unfavorable prognosis in the advanced stage for which second‐/third‐line chemotherapy is not well established.

**Case presentation:**

A 34‐year‐old woman was referred to our institution for left adrenal tumor with multiple liver metastases and tumor thrombus extending to the inferior vena cava. According to her clinical diagnosis of adrenocortical carcinoma (T4N0M1, European Network for the Study of Adrenal Tumors stage IV), we resected the left adrenal tumor and tumor thrombus. Pathological examination confirmed the adrenocortical carcinoma diagnosis. After four courses of etoposide, doxorubicin, cisplatin, and mitotane therapy, the liver metastases progressed, and we started gemcitabine, capecitabine, and mitotane therapy as second‐line chemotherapy. After 7 months, significant shrinkage of the liver metastases was observed, and they remained stable over 16 months.

**Conclusion:**

We reported a case of advanced adrenocortical carcinoma with significant shrinkage of liver metastases following gemcitabine, capecitabine, and mitotane therapy, with the effect maintained over 16 months.

**Abbreviations & Acronyms**
ACC: adrenocortical carcinoma
CT: computed tomography
DHEA‐S: dehydroepiandrosterone sulfate
EDP‐M: etoposide, doxorubicin, cisplatin, and mitotane
ENSAT: European Network for the Study of Adrenal Tumors
GC‐M: gemcitabine, capecitabine, and mitotane
IP: interstitial pneumonia
IVC: inferior vena cava
M: mitotane
MRI: magnetic resonance imaging
PET: positron emission tomography
POD: postoperative day

**Keynote message**

Significant shrinkage of liver metastases was obtained in a patient with advanced ACC by GC‐M therapy, and the effect was maintained over 16 months.

## Introduction

ACC is a rare malignant tumor. Advanced ACC is treated with mitotane monotherapy or mitotane combined with cisplatin, etoposide, and doxorubicin as first‐line chemotherapy according to the ENSAT guidelines.^1^ Second‐ and third‐line chemotherapy is not well established. Recently, gemcitabine‐based chemotherapy was reported to be well tolerated with relatively favorable responses.^2^, ^3^, ^4^ We report a patient with advanced ACC who was treated successfully with gemcitabine plus capecitabine.

## Case presentation

**Fig. 1:**
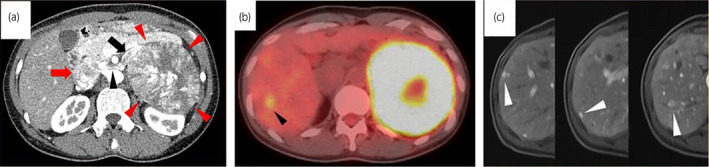
(a) Contrast‐enhanced CT showed a 110 × 100‐mm left adrenal mass (red arrowheads) and tumor thrombus extending to the IVC (black arrow: left adrenal vein, black arrowhead: left renal vein, red arrow: IVC). (b) PET‐CT showed a liver metastasis (black arrowhead), and the SUVmax was 3.7. (c) MRI showed multiple liver metastases (white arrowheads).

**Fig. 2:**
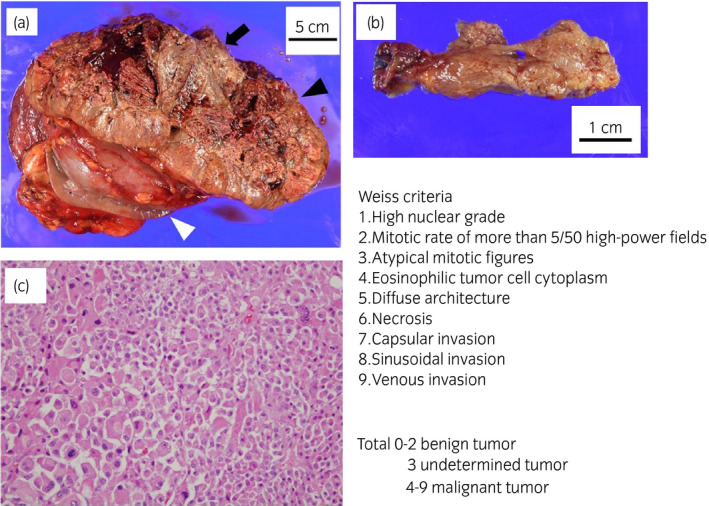
(a) Macroscopic findings of the resected tumor (black arrowhead) and left kidney (white arrowhead). The center of the tumor was necrotic (black arrow). (b) Macroscopic findings of the resected tumor thrombus. (c) Microscopic findings of the tumor. This case met 7 of 9 of the Weiss criteria (1–6 and 9).

**Fig. 3:**
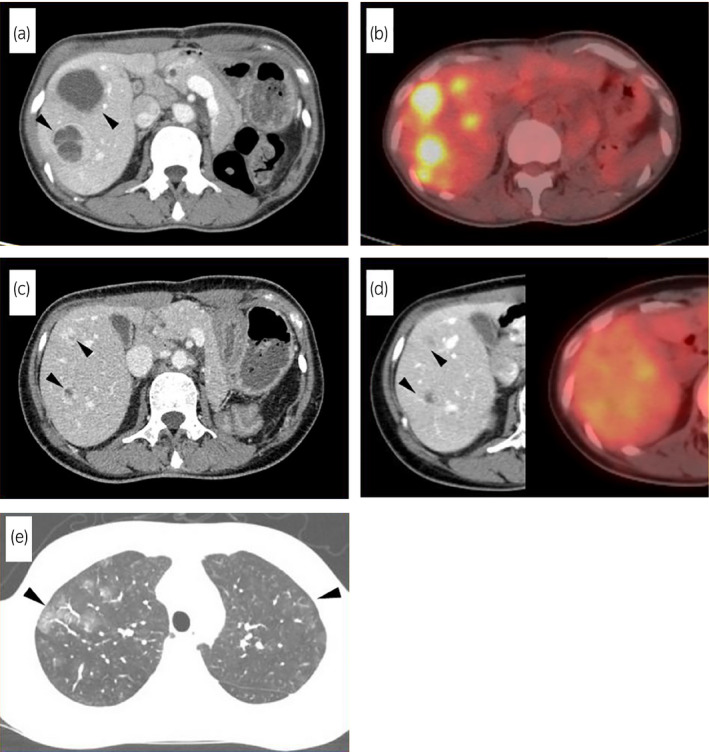
(a) At 4 months after starting EDP‐M therapy, contrast‐enhanced CT revealed that the multiple liver metastases had increased in size (black arrowheads). (b) At the same time, PET‐CT showed that the multiple liver metastases had grown larger, and the SUVmax was 8.8. (c) At 7 months after starting GC‐M, CT revealed that the multiple liver metastases had shrunk in size (black arrowheads). (d) CT and PET‐CT showed a partial response at over 12 months after starting GC‐M (black arrowheads). (e) CT showed granular shadows in the bilateral lung (black arrowheads).

**Fig. 4:**
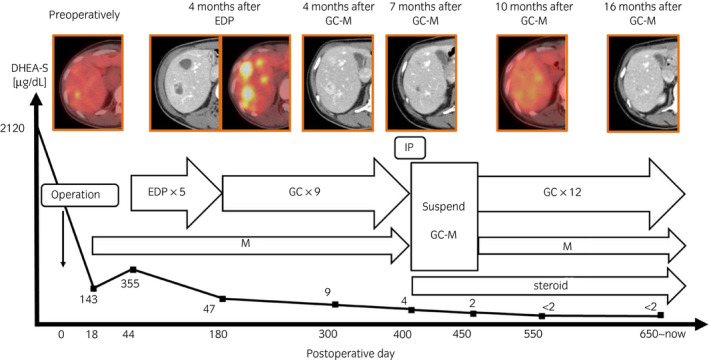
Time course of the patient’s treatment and changes in liver metastases.

A 34‐year‐old woman was referred to our institution for a 110 × 100‐mm left adrenal tumor with tumor thrombus extending to IVC, which were found on a contrast‐enhanced CT scan (Fig. 1a) for the examination of menstrual irregularity. Laboratory findings revealed high serum levels of testosterone (2.78 ng/mL) and DHEA‐S (2120 μg/dL). A PET‐CT scan (Fig. 1b) and MRI showed multiple liver metastases (Fig. 1c). We made a clinical diagnosis of ACC (T4N0M1, ENSAT stage IV). We determined that curative surgery was impossible, but we decided to resect the left adrenal tumor and tumor thrombus to reduce the risk of sudden death from the thrombus. Resection of the tumor and tumor thrombus was accomplished by combined excision of the left kidney (operative time 464 min, blood loss 2500 mL) (Fig. 2a,b). The head of the tumor thrombus reached IVC lower than the level of hepatic vein. Pathological examination confirmed the diagnosis of ACC according to the Weiss criteria (met 7 of 9 criteria) (Fig. 2c).^5^ The resected margin was negative. The Ki‐67 index was 15%. After surgery, her serum DHEA‐S decreased to 143 μg/dL. We treated the multiple liver metastases with EDP‐M (mitotane combined with etoposide 100 mg/m^2^ on days 2–4, doxorubicin 40 mg/m^2^ on days 1, and cisplatin 40 mg/m^2^ on days 3 and 4 every 4 weeks) as first‐line chemotherapy. Oral mitotane was initiated at a dose of 1.5 g/day on POD 18, and the dose was increased to 3.0 g/day on POD 31 and maintained at this dosage thereafter. EDP was initiated on POD 44. After four courses of EDP‐M, her serum DHEA‐S had decreased to 47 μg/dL, but CT and PET‐CT revealed progression of liver metastases (Fig. 3a,b). We started GC‐M (mitotane combined with gemcitabine 800 mg/m^2^ on days 1 and 8 every 3 weeks and capecitabine 1500 mg/body daily) as second‐line chemotherapy. After 9 courses of GC‐M, significant shrinkage of the liver metastases was observed (Fig. 3c), and her serum DHEA‐S decreased to 4 μg/dL (Fig. 4). At this time, she suffered from dry cough and fever. CT showed granular shadows in the bilateral lung that we determined to be gemcitabine‐induced interstitial pneumonia (Fig. 3e). We suspended GC‐M therapy and started steroid treatment (initial prednisolone dose 1 mg/kg). After 1.5 months, the interstitial pneumonia improved, and GC‐M therapy was restarted with 10 mg oral prednisolone which was continued over 8 months with gradual reduction in its dose. The other adverse events caused by GC‐M were grade 3 neutropenia, grade 3 hyponatremia, and grade 2 asthenia (grading according to the Common Terminology Criteria for Adverse Events version 5.0).^6^ Neutropenia was recovered by injection of granulocyte colony‐stimulating factor. Hyponatremia and asthenia were controlled by hydrocortisone 30 mg/day. The response to GC‐M was maintained over 16 months after initiation of the GC‐M therapy (Fig. 3d), and her serum DHEA‐S decreased to <2 μg/dL (Fig. 4).

## Discussion

ACC is a rare malignant tumor in 0.7–2 cases per 1 million population.^7^ In patients with metastatic disease, the 5‐year survival rate is below 15% and median survival time is less than 1 year.^8^ According to the results of the first randomized controlled phase III clinical trial (FIRM‐ACT), combination chemotherapy with EDP‐M represents the current first‐line treatment in advanced ACC.^1^, ^9^ There is no other systemic chemotherapy which was successfully evaluated in large randomized trials if first‐line chemotherapy fails. However, gemcitabine has been recognized as one of the most promising agents,^2^, ^10^ and the combination of gemcitabine and fluoropyrimidines, such as 5‐fluorouracil and capecitabin, is known to have a synergistic effect.^11^ Combination therapy of gemcitabine and metronomic 5‐fluorouracil or capecitabine as a second‐ or third‐line chemotherapy was first reported in 2010,^3^ and the objective response rate was 7% (2/28). A phase II study of gemcitabine‐based chemotherapy was reported in 2017^4^ in which 145 patients were analyzed retrospectively, and capecitabine was used in 132 patients. The objective response rate was 4.9%, and the respective median progression‐free and disease‐specific survival times were 12 and 40 weeks. In both reports, treatments were well tolerated. Although objective response rates were low, notably, several patients achieved long‐term disease control, and even complete responses in single patients were described.^3^, ^4^ Capecitabine is metabolized to its active form (5‐FU) by liver enzyme. This might explain the favorable response of the current case whose metastatic lesion was limited to the liver. Although the efficacy of gemcitabine‐based chemotherapy as second‐line chemotherapy is not well elucidated, it might be an option for patients who fail first‐line chemotherapy.

Usually, second‐line chemotherapy is administered with mitotane, even if first‐line chemotherapy combined with mitotane failed. Mitotane acts as a steroidogenesis inhibitor and cytostatic antineoplastic agent. Mitotane administration is often continued after failure of first‐line chemotherapy because mitotane is reported to be able to reverse the multidrug resistance mediated by *MDR‐1* expression.^12^ Although extremely rare, if long‐term objective response is achieved, careful monitoring of the side effects of mitotane is mandatory. Side effects include anorexia and nausea (88%), decreased memory and ability to concentrate (50%), diarrhea (38%), gynecomastia (50%), vomiting (23%), rash (23%), arthralgia (19%), and leukopenia (7%).^13^ Glucocorticoid replacement is also necessary in patients with long‐term mitotane use. Because mitotane increases cortisol‐binding globulin, and the cortisol bound to this protein is biologically inactive, the dose of glucocorticoid replacement is usually increased to at least twice the standard replacement dose.^1^, ^14^ Our patient took hydrocortisone at 30 mg/day.

## Conclusion

We report a patient with advanced ACC who showed a significant and prolonged response to GC‐M as second‐line chemotherapy.

## Conflict of interest

The authors declare no conflict of interest.
